# Cognitive and sensorimotor function in participants being treated for trigeminal neuralgia pain

**DOI:** 10.1186/s10194-020-01156-9

**Published:** 2020-07-17

**Authors:** Rachel O. Coats, Kirsty L. Crossley, Naomi Conlin, Jianhua Wu, Joanna M. Zakrzewska, Sue H. Pavitt, Nicholas Phillips, Mark Mon-Williams

**Affiliations:** 1 School of Psychology, University of Leeds, Leeds, LS2 9JT UK; 2 Bradford Institute for Health Research, Bradford Teaching Hospitals NHS Foundation Trust, Bradford, UK; 3 School of Dentistry, University of Leeds, Leeds, UK; 4 Royal ENT and Eastman Dental Hospitals, University College London, London, UK; 5 Leeds Teaching Hospitals NHS Trust, Leeds, UK; 6 Centre for Optics, Vision and Eye Care, University of South-Eastern Norway, Kongsberg, Norway

**Keywords:** Trigeminal neuralgia, Pain medication, Sensorimotor, Cognitive, Impairment

## Abstract

**Background:**

Trigeminal neuralgia (TN) is an orofacial condition defined by reoccurring, spontaneous, short-lived but excruciating stabbing pain. Pharmacological interventions constitute the first-line treatment for TN, with antiepileptic drugs commonly prescribed. People treated for TN pain with antiepileptic drugs describe cognitive and motor difficulties affecting activities of daily living, and report poorer quality of life. We undertook the first comprehensive objective evaluation of sensorimotor and cognitive performance in participants being treated for TN pain with antiepileptic drugs relative to age-matched controls.

**Methods:**

Participants (43 TN, 41 control) completed a battery of sensorimotor (steering, aiming and tracking) and cognitive (working memory, processing speed, inhibition) tasks.

**Results:**

The TN group performed significantly worse than controls on the sensorimotor tracking and aiming tasks and across all cognitive measures.

**Conclusions:**

The data explain why patients treated with antiepileptic drugs report impairment when conducting activities of daily living (given the need for cognitive and motor capability within most of these). The study is an important first step in: (i) ensuring there is adequate information on the impact of pharmacological treatment; (ii) identifying measures to determine optimal medication dosage and track change over time; (iii) creating an evidence base that could allow scientific justification of alternative pain treatment options for TN (e.g. the costs/benefits of surgery).

## Background

Trigeminal neuralgia (TN) is a unilateral orofacial pain condition characterised by excruciating electric shock-like pains, abrupt in onset and termination, limited to one or more distributed divisions of the trigeminal nerve [1]. TN-related pain is unpredictable in attack and remission frequency, can be triggered by innocuous stimuli (e.g. cold wind), and can greatly affect quality of life; causing sleep disturbance, depression, anxiety, impairing activities of daily living, and even suicide attempts, with an obvious impact on the individual but also on family and friends, particularly in the instance of bereavement after the suicide of a loved one [2–7].

Anti-epileptic drugs (AEDs) are the first-line treatment for TN pain, and recent European guidelines for the treatment of TN recommend carbamazepine and oxcarbazepine in the first instance [8]. AEDs usually offer initial pain relief by reducing the frequency and intensity of pain paroxysms [9–11], but their efficacy appears to decrease over time [12–15] and tolerance can be low with side-effects causing medication to be stopped or reduced to a level insufficient for pain relief [16, 17].

AEDs work by decreasing neuronal sensitivity [14] so it is unsurprising that they can disrupt function. Impaired cognitive function (e.g. reaction time, response inhibition, verbal fluency, attention, memory, mood) has been linked to AED use in patients with epilepsy [18–20], and healthy controls [21, 22]. Polypharmacy and higher dosage are related to more extreme side effects [23]. AEDs have also been linked to impaired motor function [24] including problems with balance, dizziness, gait, dexterity, ataxia, diplopia, dyskinesias, myoclonus, and parkinsonism-tremor [25, 26]. AEDs are amongst the most likely drugs to be linked with cerebellar ataxia (characterised by impaired motor coordination and problems with gait, balance, speech and eye movements [27–29]). Ataxia tends to disappear after discontinuation (although symptoms may persist, particularly with prolonged use [27]). Again, most AEDs are associated with dose-dependent risk [25, 27].

The frequently reported side effects of AEDs in the TN population have not been well quantified via objective measures, but studies suggest a profoundly negative impact. Interviews, clinical note analysis, and questionnaires have shown AEDs used for TN are often accompanied by self-reported tiredness, disturbed sleep, concentration difficulties, unsteadiness, handwriting problems, mental arithmetic struggles, and poor memory [6, 16, 30]. One study [31] measured the effects of carbamazepine on 22 patients to provide objective validation of such verbal reports. Delcker et al. [31] found postural stability, mean reaction time of tonic alertness and attention were influenced by dose. This is an important result but only captured one specific motor behaviour in a small number of patients.

In summary, studies suggest cognitive and sensorimotor side effects in TN patients taking AEDs for pain, but these verbal reports require validation and objective quantification. The current absence of quantifiable and objective measures of cognitive and sensorimotor function make it difficult to provide individuals with TN with reliable information about the potential impact of their condition. In addition, clinicians have no reliable guide to the optimum pharmacological dosage, or means of tracking how the impact of the medication changes over time. Finally, the lack of reliable evidence on the side effects of current pharmacological treatment means that justification for instigating a randomized clinical trial of surgery does not exist as the grounds for early surgical intervention are weakened if there is a satisfactory pharmacological treatment.

We aimed to compare the performance of AED-medicated individuals with TN to age-matched controls across a range of tasks in order to provide objective measures of sensorimotor and cognitive function in patients being treated for TN. Our sensorimotor measures captured critical visuomotor transformations (tracking, steering, and aiming) that underlie numerous activities of daily living. Likewise, we selected a range of cognitive tasks that captured core abilities (memory, inhibition and processing speed) widely recognized as providing the fundamental building blocks of higher order cognitive function. The weight of subjective reports of cognitive problems led us to expect the TN population to have difficulties on a number of our cognitive tasks – though it was not clear whether the nature of the difficulties would be selective or impact on all core cognitive abilities. It seemed reasonable to predict that performance on our tracking task (which taps into cerebellar function) would be detrimentally affected in the medicated TN participants compared to controls given the well-established association between AEDs and cerebellar ataxia (and the fact that patients often report postural disturbance). It was not possible to predict a priori the potential impact of AEDs on the steering and aiming tasks.

## Method

### Participants

Forty two participants with TN (28 females, 14 males, age range 24–84 years (M = 60.64, SD = 15.67) were recruited via a voluntary sampling method using associations with University College London Hospitals (UCLH) and the Trigeminal Neuralgia Association UK (TNA UK). All testing took place in the School of Psychology at the University of Leeds, in offices arranged by the TNA UK, or in a clinic through JZ at UCLH. Inclusion criteria were comprised of being over 18 years old, having a diagnosis of TN, taking AEDs for pain management, able to consent themselves personally into the study, and able to follow simple verbal instructions given in English by the researcher. Participants were excluded from the study if they had concurrent musculoskeletal problems or a neurological condition, such as Parkinson’s Disease or Arthritis, that would disrupt their ability to perform the motor tasks or confound their interpretation of the tasks by impairing their cognitive, motor or perceptual functioning. Forty six people were initially recruited and tested, but three were later excluded as they were not currently taking AED medication for pain management. One further participant had to be excluded due to suffering a pain attack during testing and was therefore unable to complete the tests.

Forty one control participants (33 females, 8 males, age range of 24–90 years (M = 60.53, SD = 15.77)) were recruited and age matched within ±2 years of the TN participants apart from one participant (aged 90) who was 6 years older than their TN age-match. Inclusion criteria consisted of being over 18 years old, having no diagnosis or history of TN, can consent themselves personally into the study, can follow simple verbal instructions given in English by the researcher. Control participants were excluded if they were taking AEDs for any reason, had concurrent health problems or neurological conditions that would disrupt their ability to perform the motor tasks or confound their interpretation of the tasks by impairing their cognitive, motoric or perceptual functioning. Recruitment was on a voluntary basis via numerous methods; the Successful Aging Panel in the School of Psychology at the University of Leeds, word of mouth, or ‘bring a friend’ scheme where they accompanied a TN participant. Forty-two control participants were initially recruited and tested, but one individual was removed because they were age-matched to the TN participant who could not complete the tasks.

All participants were free from any musculoskeletal problems that would hinder them using a handheld stylus. Ethical approval was granted from the School of Psychology Research Ethics Committee at the University of Leeds (REF: PSC-482) and accounted for all control participants and TN participants recruited via the TNA UK. NHS ethical approval (REF: 07/MRE01/38) covered all participants recruited through author JZ via UCLH. Participants provided written consent prior to taking part.

### Materials

TN participants were required to fill out a questionnaire containing 12 questions on the nature of their TN pain, their medications, and any noticeable side effects. Medications are summarised in Table 1.

**Table 1:** Type and dosage (mg) of medication by participant

			Current Medical Management						
Participant	AED Type	Therapy Type	CBZ/TEG	OXC	LTG/LAM	GBP	PGB	PHE	Non-AEDs
TN0001	1	1	800						
TN0002	3	2			200	1200			
TN0003	3	1			200				
TN0004	3	2					X	X	
TN0005	3	2		600		100			
TN0006	1	1	700						10(*A*)
TN0007	1	1	1100						20(*A*)
TN0008	2	1					25+		
TN0009	1	1	200						
TN0010	2	1				900			30(*A*)
TN0011	3	2	400			1500			
TN0012	2	1				4*			
TN0014	3	1			400				
TN0016	3	2					75	300	
TN0017	3	2		450	250		275		
TN0018	1	1		900					
TN0019	1	1	500						
TN0020	3	2	800		200				
TN0021	3	2			300			300	
TN0022	1	1		1800					
TN0023	3	2	2800				500		225(*LX*), 10(*LP*)
TN0024	1	1		500					
TN0025	1	1	12*						
TN0026	3	2		900	100				
TN0027	3	2			250–2400	900			
TN0028	2	1				900			150(*A*)
TN0029	1	1		50					
TN0030	3	1			400				
TN0032	3	1			275				
TN0033	1	1		150					
TN0034	1	1	800						
TN0036	2	1				1800			
TN0037	1	1	1200						
TN0038	1	1		1800					
TN0039	1	1	200						
TN0040	1	1	200						
TN0041	2	1				3600			
TN0042	3	2		1500		300			
TN0043	3	2		1200	400				
TN0044	3	2	300				1500		20(*A*), 180(*B*)
TN0045	1	1	600						
TN0046	3	2	700		100				

All units represent milligrams (mg) per day, unless otherwise stated. AEDs: *CBZ* Carbamazepine, *TEG* Tegretol, *OXC* Oxcarbazepine, *LTG* Lamotrigine, *LAM* Lamictal, *GBP* Gabapentin, *PGB* Pregabalin, *PHE* Phenytoin. Non-AEDs: *A* Amitriptyline, *B* Baclofen, *LX* Levothyroxine, *LP* Lisinonpril

Types of AED: 1 = Sodium only, 2 = Calcium only, 3 = Sodium and Calcium

Type of drug therapy: 1 = Monopharmacy, 2 = Polypharmacy

*Dosage unknown, participant provided number of tablets only (e.g. 12 tablets)

^+^Dosage is taken every two days

^X^Participant provided drug name but not dose

The sensorimotor battery contained tasks chosen to assess three key sensorimotor transformations that underpin a wide range of activities of daily living/motor tasks (steering, aiming and tracking). The tests were presented using the Clinical Kinematic Assessment Tool (CKAT): a tool that provides objective measures of sensorimotor performance [32]. CKAT presents interactive visual stimuli on a tablet laptop screen whilst recording participants’ kinematic responses to these stimuli. The CKAT test was implemented on a Lenovo tablet portable computer (ThinkPad Core™ M-5Y10c, screen size: 215x299mm, 1920 × 1080 pixels, 32 bit colour, 60 Hz refresh rate) with a pen-shaped stylus (140x9mm diameter) used as an input device. The CKAT software records stylus position to capture various kinematic measures (e.g. movement time, peak speed) to provide information about the accuracy and efficiency of participants’ movements. For a full overview of the CKAT software and tests see [32, 33].

The cognitive battery contained tasks chosen to assess a variety of abilities central to cognitive ability e.g. working memory, processing speed, and inhibition; widely recognised as providing the building blocks of higher order cognitive function. The tasks chosen also represented broad domains where TN patients have reported negative effects of medication [6, 34]. The tasks were completed on the tablet, with participants using finger touch to submit their responses. Headphones were used for the forward digit recall and backward digit recall tasks. Further detail on the CKAT and Cognitive Battery tasks is given below.

### Procedure

Once informed consent was gained, participants completed the questionnaire. For the sensorimotor and cognitive batteries, participants were seated at a table with the tablet screen detached from the keyboard of the laptop. This was placed in landscape orientation in front of them. Each participant completed the entire battery of tests in a single session lasting approximately 45 min. The tests were completed by all participants in the following fixed order.

#### Sensorimotor battery

**Fig. 1:**
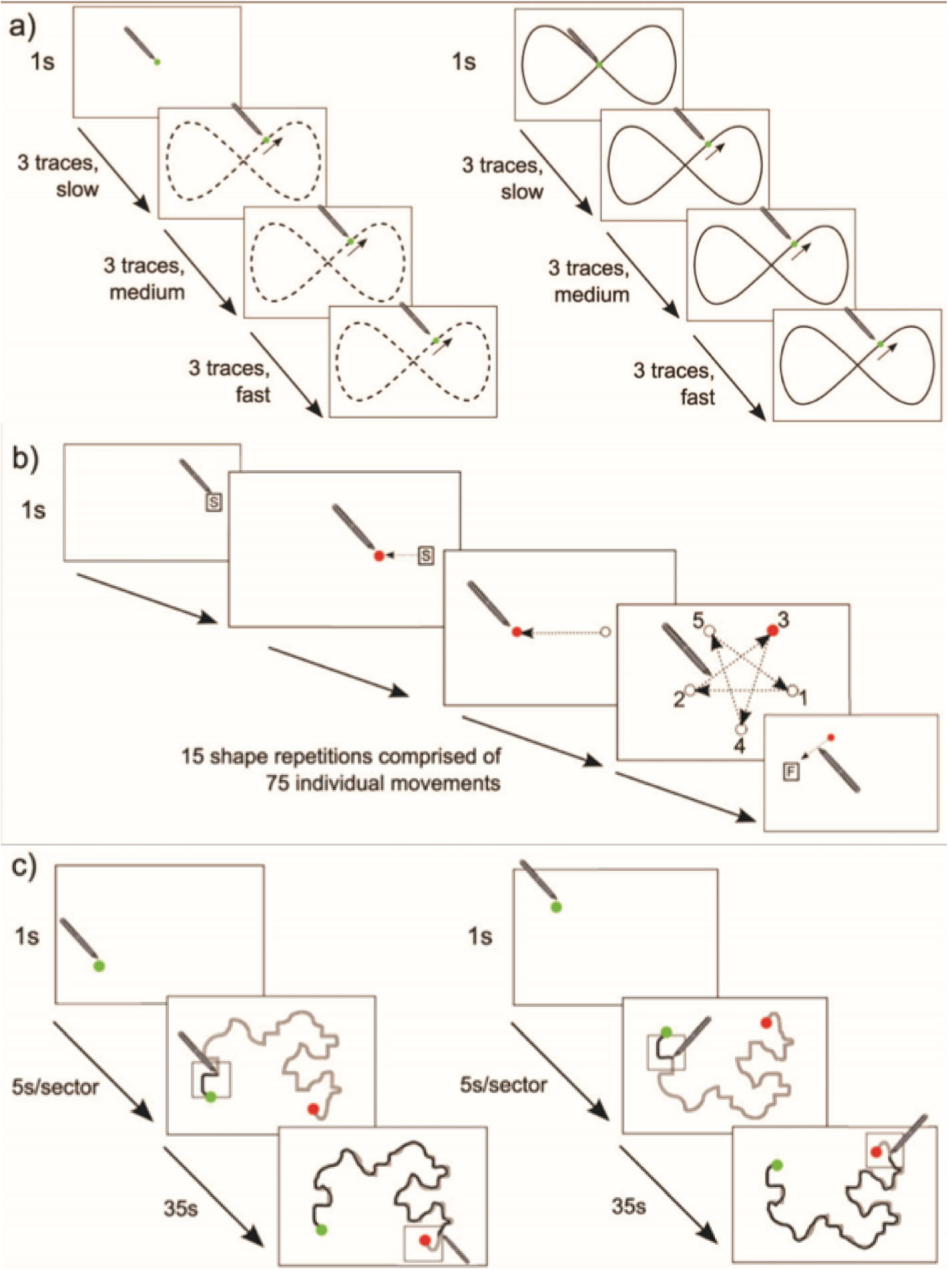
Sensorimotor Battery: **a** Tracking, **b** Aiming and **c** Steering, taken from Flatters et al. (2014). **a** Tracking: Left demonstrates the without-guide tracking trial (the dotted line indicates the pattern of movement, but participants did not see this line). Right shows the tracking trial with a guide. **b** Aiming: Arrows demonstrate direction of movement participants made but this is not visible to the participant. The 4th image shows the repeated movements that the participants made, with the numbers demonstrating which dot appeared in which sequence. **c** Steering: Left shows tracing path A and right shows tracing path B. The square box is the ‘pace’ box and the thick black lines demonstrate the pathway area that participants were expected to stay within

This battery contains three tasks (tracking, aiming and steering), lasting approximately 12–15 min in total. Figure 1 shows a graphical representation of the tasks.

##### Tracking

Participants were required to track a moving green circle displayed on the screen by keeping the tip of the stylus within the area of the green circle. The circle moved in a ‘figure-of-8’ pattern completing nine revolutions, with the speed of movement increasing after every three revolutions. The tracking task contained two consecutive trials; an unguided trial where the path can’t be seen, and a guided trial (lasting approximately 63 s each). A mean RMSE was calculated for each guide and speed condition. Lower scores indicate better performance.

##### Aiming

Participants were required to move the stylus as quickly as possible (without the stylus losing connection with the screen) from a start position to a green dot that appeared. Once the tip of the stylus reached the green circle, that target disappeared, and a new target appeared in a different location. All targets appeared at a fixed distance from each other. The participant moved from target-to-target until they had made a total of 75 discrete aiming movements, which took approximately 2–4 min. Movement time (MT) in seconds (s) (the time between arrival at 1 target dot and arrival at the next) was recorded, and the median MT for the first 50 aiming movements is reported. Shorter MTs indicate superior performance.

##### Steering

Participants were instructed to keep the tip of the stylus within a narrow pathway consisting of two parallel lines whilst moving the stylus from a ‘Start’ to a ‘Finish’ position on the screen. Speed was controlled by instructing participants to keep their stylus within a ‘pacing box’, which moved along the path sequentially in five second intervals. Participants completed two different paths (one the reverse of the other) and each took approximately 40s to complete. Path accuracy (measured as the difference between the stylus and an idealised reference path) and time were recorded. Penalized path accuracy (pPA, mm) was calculated for each path using the following formula: pPA = Path Accuracy * (1 + ((Movement time - 36)/36)). This provides a measure of accuracy that takes movement time into account to control for situations where participants do not remain within the pacing box.

#### Cognitive battery

This battery contained five tasks designed to measure simple phonological working memory, complex phonological working memory, visuospatial working memory, processing speed (PS) and inhibition. All five tasks were preceded by a practice trial. Participants were asked to interact with the visual stimuli on the screen using finger touch. The whole battery took approximately 20–30 min to complete. Tasks were always presented to participants in the same order following standardised instructions.

##### Forward digit recall (FDR)

A sequence of numbers was presented through headphones and participants were asked to recall these numbers by touching the appropriate boxes on the screen (9 boxes ordered sequentially from 1 to 9), in the order that they were originally presented. As the task progressed, the sequence length increased incrementally from three to six. There were four trials at each sequence length, with 16 trials in total over the course of the task. Reaction time (s) and response accuracy (correct or incorrect) was recorded for each item. Mean proportion correct (primary outcome variable) and mean RT (secondary outcome variable) for each sequence length is reported.

##### Backward digit recall (BDR)

Similar to FDR, but participants were asked to recall these numbers in reverse order. As the task progressed, the sequence length increased incrementally from two to five.

##### Corsi block tapping

At the start of the task nine randomly arranged blue squares were presented on the screen. A random and unique sequence of boxes flashed yellow and the participant was required to remember this order and, once the sequence had finished, respond by tapping the blue boxes in the order that they had changed colour. The difficulty was raised by increasing the sequence lengths, starting with three squares and ending with six. This task had a total of 16 trials, with four different sequences presented for each level of difficulty/sequence length. Reaction time (s) and response accuracy (correct or incorrect) was recorded for each item. Mean proportion correct (primary outcome variable) and mean RT (secondary outcome variable) for each sequence length is reported.

##### Processing speed (PS)

Red circles, red triangles and blue circles were presented on the screen. The participants were asked to identify how many red circles were present on the screen and respond by tapping the box located at the bottom of the screen containing the correct number. Participants were requested to carry out each of the 18 trials as quickly and accurately as possible. Reaction time (s) and response accuracy (correct or incorrect) was recorded for each item. Mean RT for correct trials is reported.

##### Inhibition (flankers)

The participants were presented with a line of five arrows in the centre of the screen. They were required to identify the direction of the middle arrow in two different conditions, where the remaining four arrows were either pointing in the same (congruent) or opposite (incongruent) direction as the middle arrow. Participants were required to answer as quickly and accurately as possible. Mean reaction time (s) for the congruent and incongruent trials is reported.

### Statistical analyses

This was a mixed research design with various independent (IV) and dependent (DV) variables depending on the task (see Table 2 for details). The statistical software package JASP [35] was used to explore the CKAT battery and Cognitive battery RT data, and SPSS [36] used for the Cognitive battery proportion correct analyses. For all tasks, group (TN/Control) was treated as a between subjects variable and all other IVs as within subjects variables.

**Table 2:** Experimental Tasks and Independent (IV) and Dependent (DV) Variables

Task	IV	DV
Tracking	Group/ Guide/ Speed	RMSE
Aiming	Group	RT (s)
Steering	Group/ Pathway	pPA (mm)
BDR	Group/ Sequence Length	Proportion Correct / RT (s)
FDR	Group/ Sequence Length	Proportion Correct / RT (s)
Corsi	Group/ Sequence Length	Proportion Correct / RT (s)
Inhibition	Group/ Congruency	RT (s)
PS	Group	RT (s) for correct trials

*BDR* Backward Digit Recall, *FDR* Forward Digit Recall, *PS* Processing Speed, *RMSE* Root Mean Square Error, *RTs* Reaction Time, *pPA* Penalised Path Accuracy

#### CKAT battery and cognitive battery reaction time data

Mixed ANOVAs were used to explore Tracking, Steering, FDR RT, BDR RT, Corsi RT, and Flankers RT. Aiming and Processing speed contained only one independent variable (Group) for which an independent t-test and Mann-Whitney test were used (the latter due to deviations from normality). Outliers for the CKAT battery tasks were removed if they were two standard deviations away from the mean (24 trials in total). Four further trials were removed due to technical failures. Where Mauchly’s test of sphericity was violated, a Greenhouse-Geisser correction was applied. When Levene’s test for homogeneity of variance was violated (unsurprisingly often given the nature of the variability of the TN sample) this is reported, and the data were transformed using a reciprocal transform to achieve homogeneity. The data displayed in the figures is the original pre-transform data. Finally, where pairwise comparisons were used to explore significant main effects, a Bonferroni Holm correction was applied, and only interactions involving group, and therefore relevant to the specific research question, are explored for the sake of brevity.

#### Cognitive battery proportion correct

The proportion correct data (for FDR, BDR and Corsi) could not be transformed to correct for significant deviations from homogeneity of variance. For this reason, we categorised participants as either high or low performers using the mean performance across all participants for each sequence length as the cut off between high and low performance. We then used a Chi Squared χ^2^ test to check for associations between group and performance (high or low). The data displayed in the figures is the original pre-categorisation data.

#### Additional analyses

Finally, we took the opportunity to conduct some post-hoc analyses on the effects of monopharmacy (*n* = 15) vs polypharmacy (*n* = 27) and AED type within the medicated TN group (see Table 1 for details). We compared participants taking sodium blockers only (*n* = 17) to those taking drugs that blocked both calcium and sodium (*n* = 19). There were too few people taking calcium blockers alone (*n* = 6) to include as a group. Once more, group was treated as an independent variable and the rest of the design and all conducted analyses remained the same. We also tested for an association between dose and performance on each task. We calculated the total quantity of AED medication (*mg*) for each participant in the TN group and correlated this with task performance.

## Results

### Sensorimotor battery

#### Tracking

**Fig. 2:**
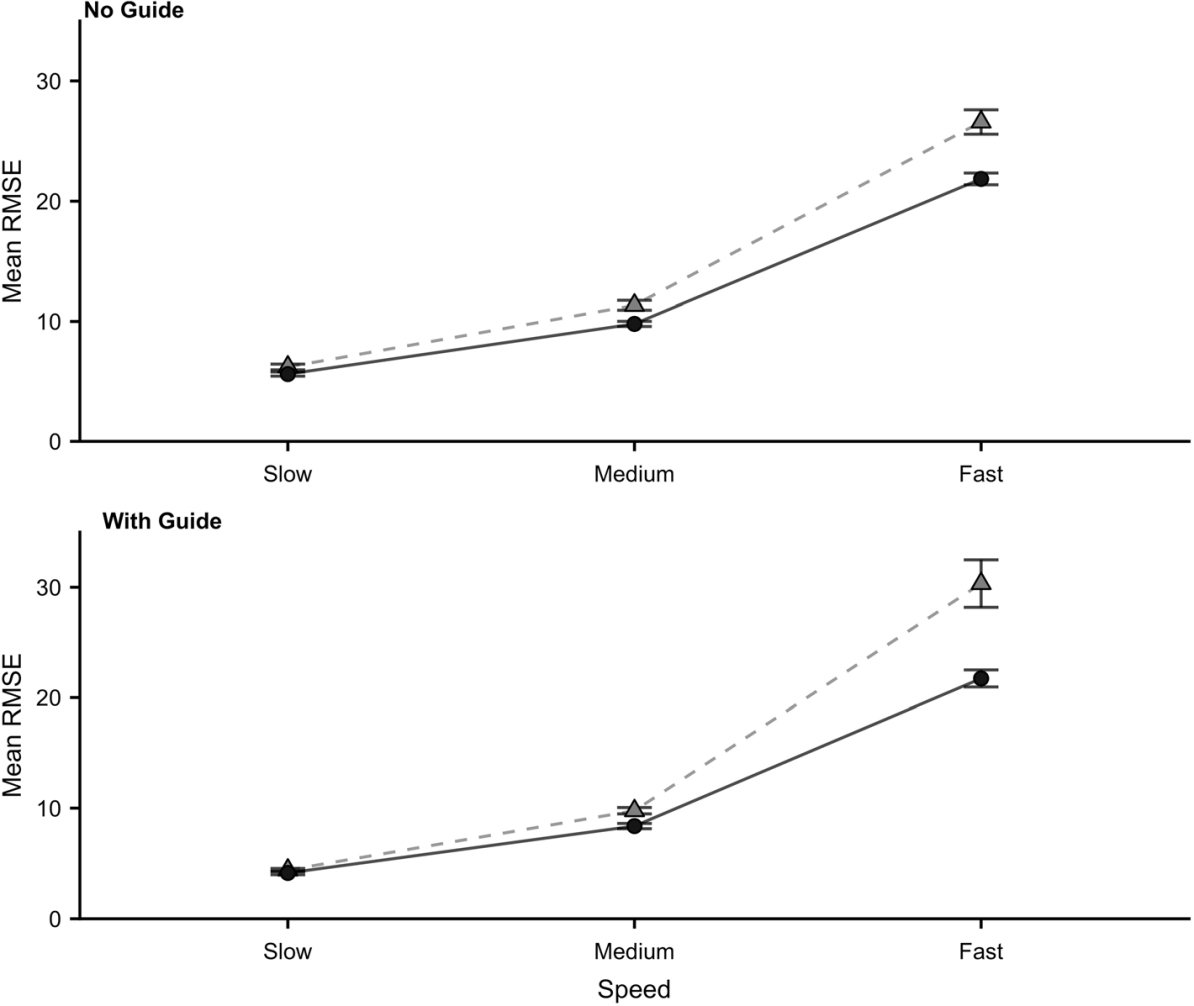
Tracking*.* Mean root mean square error (RMSE) for both groups (Control = circles, TN = triangles) across all speeds. The top plot shows the No Guide condition and the bottom plot the With Guide condition

A 2 × 2 × 3 mixed ANOVA was conducted to analyse the effect of group (TN/Control), guide (with guide/no guide) and speed (slow/medium/fast) on RMSE. A reciprocal transform was used to achieve homogeneity of variance and a mixed ANOVA was performed on these data. Raw scores pre-transform can be seen in Fig. 2. The Levene’s test remained significant for the ‘Fast With Guide’ condition. Data were available from 36 TN participants and 36 Controls. A main effect of group emerged [*F* (1,70) = 7.44; *p* < 0.01, *η*^2^ = 0.10] with RMSE being significantly higher in the TN group. Main effects for Guide [*F* (1,70) = 185.99; *p* < 0.01, *η*^2^ = 0.73] and Speed [*F* (1.11,78.61) = 1915.63; *p* < 0.001, *η*^2^ = 0.96] emerged, with RMSE significantly increasing (p < 0.001) as the speed of the dot increased. There was also a Speed x Guide interaction [*F* (1.25, 87.52) =187.44; *p* < 0.001, *η*^2^ = 0.73].

#### Aiming

An independent t-test was used to test for a difference in movement time (s) between the two groups. Data were included from 40 control and 40 TN participants. Movement Time (MT) was significantly longer for the TN participants (mean = 1.33 s, SD = 0.18) than the controls (mean = 1.24 s, SD = 0.20) [*t* (78) = 2.10, *p* < 0.05, *Cohen’s d* = − 0.47].

#### Steering

A 2 × 2 mixed ANOVA was conducted to analyse the effects of group (TN/Control) and path (path A and B) on penalised path accuracy. Data were included from 39 control and 40 TN participants (Control path A: mean = 1.33 s, SD = 0.54; Control path B: mean = 1.23 s, SD = 0.45; TN path A: mean = 1.40s, SD = 0.54; TN path B: mean = 1.31 s, SD = 0.49)*.* No main effects or interactions emerged [Group *F* (1,77) = 0.561, *p* = 0.456, *η*^2^ = 0.007; path *F* (1,77) = 2.849, *p* = 0.1, *η*^2^ = 0.036; Group x path *F* (1,77) = 0.001, *p* = 0.970, *η*^2^ = 0.00].

### Cognitive battery

#### Forward digit recall (FDR)

##### Proportion correct

**Fig. 3:**
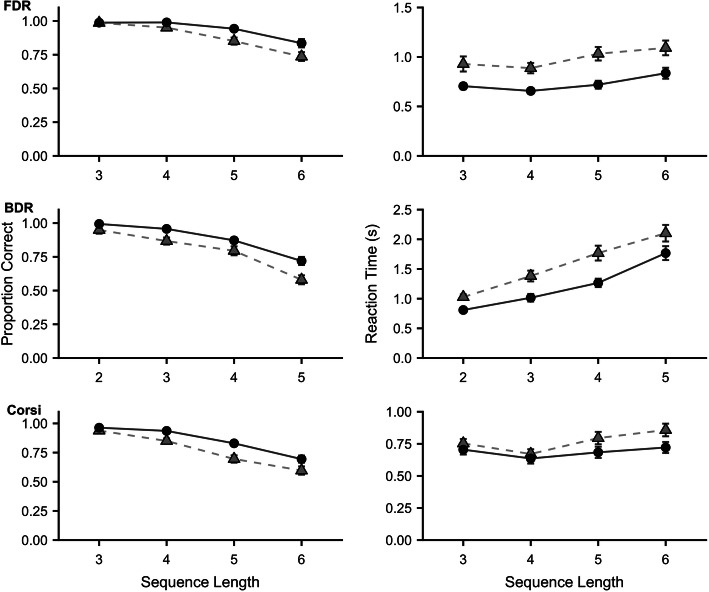
Forward Digit Recall (FDR); Backward Digit Recall (BDR); Corsi Block Tapping. Mean proportion correct (column 1) and mean reaction time (column 2) for both groups (Control = circles, TN = triangles) across all sequence lengths

A *χ*^*2*^ test was used to test for an association between participant group (TN/Control) and obtaining a high proportion correct score. Participants were at ceiling for trials with a sequence length of three so these cannot be analysed. For trials with a sequence length of four there was no significant association between the participant group (TN/Control) and whether they were likely to get a high score [*χ*^*2*^ (1) = 1.89, *p* = 0.14, odds ratio = 2.25]. For trials with a sequence length of five or six there was a significant association between the group the participants were in (TN/Control) and whether or not they were likely to get a high score [*length 5*: *χ*^*2*^ (1) = 7.623, *p* < 0.01, *length 6*: *χ*^*2*^ (1) = 5.529, *p* < 0.05]. Based on the odds ratio, the odds of getting a high score were 3.97 (sequence length 5) and 2.93 (sequence length 6) times higher if the person was a Control participant than a TN participant. Raw scores pre categorisation can be seen in Fig. 3.

##### RT

For the reaction time (RT) data a reciprocal transform was used to achieve homogeneity of variance and a 2 × 4 mixed ANOVA performed on these data to analyse the effect of group (TN/Control), and sequence length (3,4,5,6) on RT. Raw mean RT scores pre-transform can be seen in Fig. 3. A significant main effect of group emerged [*F* (1,81) = 17.94, *p* < 0.001, *η*^2^ = 0.181] with RTs longer for the TN group than Control group. There was also a main effect of sequence length [*F* (2.51, 203.64) = 14.96, *p* < 0.001, *η*^2^ = 0.15] caused by significant differences between all sequence lengths (*p* < 0.05) apart from 3 and 4 (*p* = 0.09). No group x sequence length interaction emerged [*F* (2.51, 203.64) = 2.09, *p* = 0.114, *η*^2^ = 0.02].

#### Backward digit recall (BDR)

##### Proportion correct

Participants were at ceiling for trials with a sequence length of two, so these cannot be analysed. For trials with a sequence length of four there was no significant association between participant group (TN/Control) and whether they were likely to get a high score [*χ*^*2*^ (1) = 3.046, *p* = 0.064, odds ratio = 2.25]. For trials with a sequence length of three and five there was a significant association between the group the participants were in (TN/Control) and whether or not they were likely to get a high score [*length 3*: *χ*^*2*^ (1) = 3.013, *p* < 0.05, *length 5*: *χ*^*2*^ (1) = 6.467, *p* < 0.05]. Based on the odds ratio, the odds of getting a high score were 3.23 (sequence length 3) and 3.17 (sequence length 5) times higher if the person was a control participant than a TN participant. Raw scores pre categorisation can be seen in Fig. 3.

##### RT

For the reaction time (RT) data a reciprocal transform was used to achieve homogeneity of variance and a 2 × 4 mixed ANOVA performed on these data to analyse the effect of group (TN/Control), and sequence length (2,3,4,5,) on RT. Raw mean RT scores pre-transform can be seen in Fig. 3. A significant main effect of group emerged [*F* (1,81) = 18.41,*p* < 0.001, *η*^2^ = 0.185] with RTs longer for the TN group than Control group. There was also a main effect of sequence length [*F* (2.71, 219.78) = 110.98, *p* < 0.001, *η*^2^ = 0.57] caused by significant differences between all sequence lengths (all p < 0.001). A group x sequence length interaction emerged [*F* (2.71, 219.78) = 2.96, *p* < 0.05, *η*^2^ = 0.015] supporting what can be seen in the figure: that group differences increased as sequence length increased.

#### Corsi block tapping (Corsi)

##### Proportion correct

For trials with a sequence length of three there was no significant association between the group a participant was in (TN/Control) and whether they were likely to get a high score [*χ*^*2*^ (1) = 0.863, *p* = 0.25, odds ratio = 1.59]. For trials with a sequence length of four, five and six there was a significant association between the participant group (TN/Control) and whether or not they were likely to get a high score [*length 4*: *χ*^*2*^ (1) = 5.23, *p* < 0.05, *length 5*: *χ*^*2*^ (1) = 6.37, *p* < 0.01, *length 6*: *χ*^*2*^ (1) = 3.49, *p* < 0.05]. Based on the odds ratio, the odds of getting a high score were 2.81 (sequence length 4), 3.12 (sequence length 5), and 2.30 (sequence length 6) times higher if the person was a control participant than a TN participant. Raw scores pre categorisation can be seen in Fig. 3.

##### RT

A 2 × 4 mixed ANOVA was conducted to analyse the effect of group (TN/Control), and sequence length (2,3,4,5,) on RT. Mean RT scores can be seen in Fig. 3. Reaction times were longer for the TN group than the control group but the main effect failed to reach conventional levels of significance [*F* (1,81) = 3.959, *p* = 0.09, *η*^2^ = 0.035]. There was also a main effect of sequence length [*F* (2.61, 211.41) = 7.69, *p* < 0.001, *η*^2^ = 0.085] caused by differences between sequence lengths of 3 and 4, 4 and 5 and 4 and 6 (all *p* < 0.01). No group x sequence length interaction emerged [*F* (2.61, 211.41) = 1.44, *p* = 0.231, *η*^2^ = 0.016].

### Processing speed

A Mann-Whitney U test revealed a significant difference in RTs between the groups [*U* = 465.00, *p* < 0.001, rank biserial correlation = − 0.460] with RTs being longer for the TN group (mean = 3.69 s, SD = 0.86) compared to Controls (mean = 3.14 s, SD = 0.74).

### Inhibition (flankers)

A reciprocal transform was used to achieve homogeneity of variance and a 2 × 2 mixed ANOVA performed on these data to analyse the effect of group (TN/Control), and condition (congruent/incongruent) on RT. A significant main effect of group emerged [*F* (1,81) = 11.45, *p* < 0.001, *η*^2^ = 0.124] with raw RTs for the TN group (mean = 0.12 s, SD = 0.40) being longer than those for the Control group (mean = 0.86, SD = 0.24). There was also a main effect of condition [*F* (1,81) = 43.70, *p* < 0.001, *η*^2^ = 0.346] with RTs being longer for incongruent (mean = 1.03 s, SD = 0.37) compared to congruent trials (mean = 0.95, SD = 0.27). There was no group × condition interaction [*F* (1,81) = 1.51, *p* = 0.223, *η*^2^ = 0.012].

### Additional analyses

We first explored monopharmacy versus polypharmacy. We found no main effect of group for any of the sensorimotor measures (Aiming, Tracking, and Steering). There were no differences between groups on reaction time for Processing Speed or Inhibition tasks. For FDR, BDR and Corsi (our working memory tests) we found no associations between group and proportion correct but we did find a significant main effect of group on reaction time for both FDR [*F* (1,40) = 4.198, *p* < 0.05, *η*^2^ = 0.095] and BDR [*F* (1,40) = 4.424, *p* < 0.05, *η*^2^ = 0.1]. In both cases RTs were longer for those undergoing polypharmacy (FDR mean = 1.146, BDR mean = 1.74) compared to monopharmacy (FDR mean = 0.896, BDR mean = 1.475). We next explored whether there would be any differences between groups taking different types of AED. We found no main effects of group on any of our measures. Finally, we tested for an association between dose and performance and found a significant association on the Backward Digit Recall task when sequence length was highest (sequence length 5) with higher doses associated with poorer performan*ce* [*r* = 0.321; *p* = 0.049].

## Discussion

This study sought to quantify the cognitive and sensorimotor side effects experienced by individuals being treated for TN with AEDs. Performance on a battery of sensorimotor and cognitive tasks of participants with a diagnosis of TN who were currently undergoing pain management in the form of AED medication was compared to that of age-matched controls. Results from the sensorimotor battery showed that the TN participants (taking AEDs) were significantly worse at tracking a moving object than Controls (Tracking task) and were slower than controls to move to a target when it appeared on screen (Aiming task). Our TN group were no worse than controls in terms of accuracy when steering through a defined path (Steering task). Findings from the cognitive battery point towards clear impairments in the TN group. Participants with TN produced longer reaction times across all the working memory tasks (FDR, BDR and Corsi). In addition, the participants in the TN group were ~ 2–4 times less likely to be “high scorers” (where the mean proportion correct across the whole sample was used to categorise participants into high or low scorers) than participants in the Control group. Finally, participants in the TN group were significantly slower than controls in the Processing Speed task, and significantly less able than controls to inhibit unwanted responses in the Inhibition (Flankers) task.

The poor tracking performance on our sensorimotor battery shown by participants with TN is completely consistent with the extensive literature on AEDs and disrupted cerebellar function [24, 25, 27–29]. This, in addition to the reduced performance on the Aiming task, corroborates the accounts often provided by medicated people with TN: that they are experiencing sensorimotor impairments. The tasks in our battery reflect core components of real-world skills such as driving, hand-writing, and controlling posture, and our results can therefore go some way to explaining why people with TN have reported difficulties with such activities in the past [6, 17], and are in line with the work of Delcker et al. [31] who quantified variations in postural stability in medicated patients with TN. As with the sensorimotor battery, our findings with regards to performance on our cognitive battery clearly triangulate evidence from self-report and qualitative studies [6, 14, 17, 30], suggesting that patients with TN experience cognitive impairment as a result of pain medication. Our results are also consistent with studies reporting the negative cognitive side effects caused by AEDs in other populations [18–23].

Our sensorimotor and cognitive measures were designed to capture core abilities that underpin a range of motor and higher-order cognition tasks. The data can therefore explain why patients being treated for TN report difficulties with a range of motor and cognitive tasks. Moreover, a number of activities of daily living require an interaction between the motor and cognitive system (e.g. making a cup of tea requires someone to remember the sequence of events that result in a palatable hot beverage but also relies on the motor ability that results in the requisite actions unfolding across space and time). The Cognition Action Interaction Theory [37] suggests that these somewhat different systems (i.e. motor and higher-order cognition) are intrinsically intertwined and impact on one another. This explains why patients being treated for TN are so severely disabled – underlying difficulties with both the sensorimotor and cognitive systems will create problems across a multitude of daily living activities.

The existing research literature suggests that polypharmacy is linked to more extreme side effects [23]. We conducted a post-hoc analysis to explore whether there was any evidence for polypharmacy effects in the TN population. The results showed that performance on our phonological working memory tasks (FDR and BDR) was affected detrimentally to a greater extent in those undergoing polypharmacy compared to monopharmacy. However, AED dosage is also linked to more extreme side effects and we could not rule out the possibility that the polypharmacy was related to dosage. In fact, when we examined associations between dose and performance a significant correlation emerged for the BDR task at the longest sequence length, where higher dose was associated with poorer performance. Despite this providing only limited evidence for the effect of dose, we don’t think it can be ruled out due to the nature of our sample (it was varied, and some participants were unable to report exact dose). A larger clinical trial is required. We also conducted a post hoc analysis to examine the impact of AED type (‘sodium’ versus ‘calcium+sodium’ blockers) but found no group differences. It is not possible to draw conclusions from this failure to reject the null hypothesis, but the finding does indicate that all AEDs have the potential to cause side effects of the type reported within this study.

If drug treatment becomes ineffective, or the side effects of medication are too severe, then surgery can offer an alternative treatment option [8, 38]. Treatments are categorized as either destructive (involving intentional destruction of sensory nerve function) or non-destructive (involving decompression of the trigeminal nerve). Surgical interventions (such as gamma knife) hold great potential for treating the pain associated with TN whilst avoiding the potential side effects of medication. Unfortunately, there are few robust clinical trials of surgical interventions [8, 39], and the evidence base to support adoption of surgical treatment at the point of TN diagnosis is lacking. One major problem is that the side effects of medications for TN (such as AEDs) have not been measured robustly or objectively (refer to Introduction). It is our hope that the study presented here can start to address this problem by providing more detail about the side effects patients can expect. In addition, even if surgery proves to be the best option, details on AED side effects remain crucial because pain may recur, and many patients remain on medication even after surgery because the pain relief can be delayed for a few months [8] and, in the absence of a robust RCT, patients (and clinicians) fear that the pain may return [40].

It is not possible to rule out the possibility that the poor performance shown by the participants with TN was due to factors other than their pharmacological treatment. In order to establish this unequivocally, we would have needed to ask participants to stop taking their medication. The ethical justification for this was absent when we ran the study as we had not yet established that there were any objective deficits in sensorimotor or cognitive function. The present study would provide such a justification for any scientists who felt that there were feasible reasons for supposing that the decrements in sensorimotor and cognitive function were due to other factors, and such a study should include further information on participant clinical characteristics such as TN duration and history of other diseases. Nevertheless, we would argue that it is likely that the reported effects are due to the medication. First, our findings are completely consistent with a large body of evidence suggesting that AEDs cause deficits in sensorimotor and cognitive function. Second, our findings are in accord with the reports of patients with TN who ascribe these deficits to the medication. Third, none of our participants were experiencing an attack of pain during the testing (thereby ruling out the symptoms of TN as a causal factor in the observed deficits). Finally, it is very difficult to explain why problems with the trigeminal nerve would cause deficits of the type we have identified in the present study.

## Conclusions

We undertook the first comprehensive objective evaluation of sensorimotor and cognitive performance in participants being treated for TN pain with antiepileptic drugs relative to age-matched controls and found that the TN group performed significantly worse than controls on the sensorimotor tracking and aiming tasks and across all cognitive measures. We suggest that our findings provide strong support for research into alternative approaches to pain management for people with TN. Whilst the cost-benefit ratio for AEDs might be justifiable for epilepsy, suppressing brain activity to treat TN pain is potentially sub-optimal, and the current study together with the existing body of research justifies exploration of alternative options, either in the form of surgery or non-AED pharmacological interventions that come with fewer side effects. It is our hope that this work will lay the foundations for large-scale studies comparing different treatments (both pharmacological and surgical). Our results have the potential to: (i) help researchers find more effective pain management strategies; (ii) provide critical information to patients and clinicians (thereby allowing them to make effective treatment choices); and (iii) ultimately reduce or even remove the side effects experienced by people being treated for TN.

## Data Availability

The datasets used and/or analysed during the current study are available from the corresponding author on reasonable request.

## Abbreviations

ADL: Activities of daily living
AEDs: Antiepileptic drugs
BDR: Backward digit recall
CKAT: Clinical kinematic assessment tool
FDR: Forward digit recall
MT: Movement time
pPA: Penalised path accuracy
PS: Processing speed
RMSE: Root mean square error
RT: Reaction time
TN: Trigeminal Neuralgia
TNA UK: Trigeminal Neuralgia Association UK
UCLH: University College London hospital
